# Tomato phyE Is Required for Shade Avoidance in the Absence of phyB1 and phyB2

**DOI:** 10.3389/fpls.2016.01275

**Published:** 2016-09-16

**Authors:** Amanda Schrager-Lavelle, Leslie A. Herrera, Julin N. Maloof

**Affiliations:** Department of Plant Biology, University of California, DavisDavis, CA, USA

**Keywords:** elongation, internode, phytochrome, shade avoidance, *Solanum lycopersicum*, tomato

## Abstract

The phytochrome (phy) family of red and far-red photoreceptors provides plants with critical information about their surrounding environment and can signal downstream developmental and physiological changes. Neighboring plants compete for limited light resources, and their presence is detected by the phytochrome photoreceptors as a reduced ratio of red: far-red light. One common response to shade is increased elongation of petioles and internodes to compete with their neighbors. While the phytochrome family, phyB in particular, has been well studied in Arabidopsis, information about the other phytochrome family members is limited, especially in sympodial crop plants such as tomato, that have a very different architecture from that of the model plant. To study the tomato phytochrome family we took advantage of several existing mutants and generated an artificial miRNA (amiRNA) line to target *SlPHYE*, the remaining phytochrome B subfamily member with no currently available mutant line. Here, we characterize internode elongation and shade avoidance phenotypes of the *SlPHYE* amiRNA line (*PHYE* amiRNA). In addition, higher order phytochrome subfamily B mutants were generated with the *PHYE* amiRNA line to investigate the role of SlphyE within the phyB subfamily. We find that the *PHYE* amiRNA line has no detectable phenotype on its own, however in higher order combinations with *SlphyB1* and/or *SlphyB2* there are notable defects in shade avoidance. Most notably, we find that the triple mutant combination of *SlPHYE* amiRNA, *SlphyB1*, and *SlphyB2* has a phenotype that is much stronger than the *SlphyB1 SlphyB2* double, showing constitutive shade avoidance and little to no response to shade. This indicates that SlphyE is required for the shade avoidance response in the absence of SlphyB1 and SlphyB2.

## Introduction

Plant growth and development are dependent not only on internal signals, but environmental factors as well. Light is essential for the growth and development of all plants; not only because light is the essential energy source, but also because light signals provide plants with information about their surrounding environment. Plants monitor changes in the quality and direction of light to optimize germination, growth, and development, and to allow optimal capture of light for photosynthesis (Franklin and Whitelam, 2005). Because light is vital for plants, shade poses a significant challenge. Plants detect foliar shade (which has a low ratio of red to far-red light) through the red and far-red light phytochrome photoreceptors. When shade is detected, plants are able to undergo a developmental response, termed shade avoidance (Smith and Whitelam, 1990). This response includes internode and petiole elongation, early flowering, and an increase in apical dominance to allow the plant to better compete with neighbors for light resources.

The plant species *Arabidopsis thaliana* provides a powerful genetic and molecular tool for studying shade avoidance, but with its rosette architecture, research on this species is ultimately limited because it does not have substantial vegetative internodes. This is significant because internode elongation is the hallmark of shade avoidance in most plants and little is known about genes important for shade-regulated internode elongation. Studying shade avoidance in tomato provides an excellent opportunity to overcome this limitation as tomato has vegetative internodes that are strongly shade responsive. While the phytochrome family is well-known to play a significant role in shade avoidance, it is unknown which of these phytochromes are important for internode elongation. It is also not fully known how well the Arabidopsis phytochrome family serves as a model for other organisms, especially those with a very different architecture than Arabidopsis.

**Figure 1:**
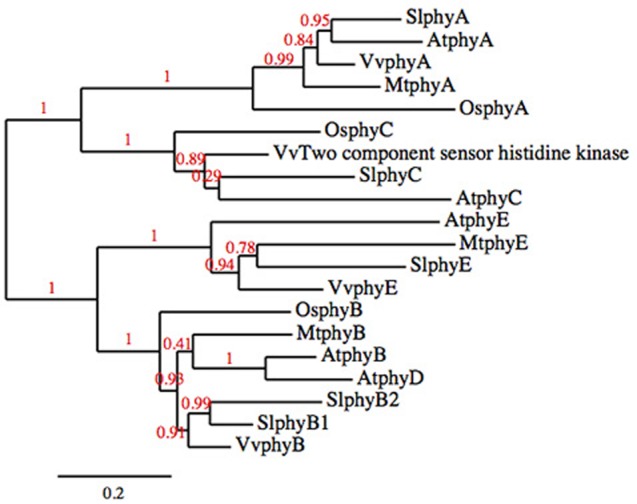
**Phylogenetic tree of the phytochrome families of tomato, Arabidopsis, rice, grape, and ***Medicago truncatula*****.

Phytochrome photoreceptors are present in all plants; in flowering plant lineages there are two phytochrome subfamilies, *PHYA/PHYC*, and *PHYB. PHYB* has undergone several independent duplications such that not every *PHYB* subfamily member is present in every lineage (Clack et al., 1994; Devlin et al., 1999; Mathews, 2010). The phytochrome family of both Arabidopsis and tomato consist of five genes, *PHYA, PHYC*, and three members of the *PHYB* subfamily, *PHYE* and two paralogs of *PHYB* that have arisen through independent duplication (*PHYB* and *PHYD* in Arabidopsis, *PHYB1* and *PHYB2* in tomato; Clack et al., 1994; Alba et al., 2000; Figure 1).

Previous research in Arabidopsis showed the phyB family is largely responsible for shade avoidance with phyB as the dominant photoreceptor and phyD and phyE playing minor and/or redundant roles (Franklin et al., 2003). Interestingly, this work and that of others (Weller et al., 2000) shows a different role for the tomato phytochrome family members as compared to Arabidopsis. In order to investigate the role of the tomato *PHYB* family members, we took advantage of the previously characterized *phyA* (Lazarova G. I. et al., 1998b), *phyB1* (Lazarova G. I. et al., 1998a), and *phyB2* (Weller et al., 2000) null mutants and generated an artificial microRNA (amiRNA; Alvarez et al., 2006; Schwab et al., 2006; Warthmann et al., 2008) to knock down expression of *PHYE* mRNA (*PHYE* amiRNA). In this manuscript, we characterize the role of phyE in both internode elongation and shade avoidance and demonstrate that phyE is required for shade avoidance in the absence of phyB1 and phyB2.

## Materials and methods

### Plant material

Wild-type cultivar Moneymaker (accession LA2706) and phytochrome mutants in the Moneymaker background; *phyA* (*fri-1*, accession LA3809), *phyB1* (*tri-1*, accession LA4357), *phyB2* (accession LA4358), *phyB1*/*phyB2* (accession LA4364), and *phyA/phyB1/phyB2* (accession LA4366) seed were obtained from the UC Davis/C.M. Rick Tomato Genetics Resource Center, maintained by the Department of Plant Sciences, University of California, Davis, CA.

### *PHYE* amiRNA generation

The *PHYE* amiRNA was designed using the WMD-3 Web MicroRNA designer (http://wmd3.weigelworld.org/cgi-bin/webapp.cgi) and generated by modifying the pRS500 plasmid (Addgene, plasmid #22846) through PCR (see Table 1 for *PHYE* amiRNA generation primers and PCR conditions) to generate the amiRNA precursor fragment of interest following the WMD-3 Web MicroRNA protocol (http://wmd3.weigelworld.org/downloads/Cloning_of_artificial_microRNAs.pdf, Schwab et al., 2006). This PCR product was cloned into expression vector pMDC32 behind a 2x 35S promoter. The pMDC32 vector contains a 35S:HPT hygromycin resistance gene for plant selection (Curtis and Grossniklaus, 2003). The pMDC32 vector containing the *PHYE* amiRNA was transformed into Agrobacterium strain GV3101 via heat shock transformation.

**Table 1:** **Primer sequences and PCR/ qPCR conditions for genotyping, *PHYE* amiRNA generation, and expression analysis**.

**Genotyping primers**		
phyA dCAPS F (EcoNI cuts WT)	TAACTGAATACACCATTCCCTTAACC	47⋅C/45 s
phyA dCAPS R (EcoNI cuts WT)	ATAATCGCTCTATAGTCACC	WT 215/20; *phyA* 235
phyB1 dCAPS F (HinF1 cuts WT)	CTAAAATTCAAAGAGGAGGTCAGATT	58⋅C/20 s
phyB1 dCAPS R (HinF1 cuts WT)	GAAGGGGTAAAAAGGGTCCTAA	WT 172/20; *phyB1* 192
phyB2 F WT specific	CCCTTTTTCCTTTTCTGACC	62⋅C/45 s
phyB2 R non-specific	GACAATATTGAGGATGGGTA	WT 513; *phyB2* absent
phyB2 F mutant specific	GTCTTGATTTCGTCTGGA	66.5⋅C/45 s
phyB2 R non-specific	GACAATATTGAGGACGGGAGAGTT	WT absent; *phyB2* 452
phyE amiRNA F	GCTCGGACGCATATTACACA	58⋅C/30 s
phyE amiRNA R	ACCATGATTACGCCAAGCTC	WT absent; *PHYE* amiRNA 493
positive control F	TGATGTTGATGGGCAGGTTA	58–67⋅C/30 s
positive control R	CACTCAGAACACCAGCCAAA	676
Genotyping primers for phyA, phyB1, and phyB2 (Weller et al., 2000).		
***PHYE*** **amiRNA generation primers**		
I miR-s	GATAAATCTGACAGAAGACGCTGTCTCTCTTTTGTATTCC	
II miR-a	GACAGCGTCTTCTGTCAGATTTATCAAAGAGAATCAATGA	
III miR^*^s	GACAACGTCTTCTGTGAGATTTTTCACAGGTCGTGATATG	
IV miR^*^a	GAAAAATCTCACAGAAGACGTTGTCTACATATATATTCCT	
Oligo A	CTGCAAGGCGATTAAGTTGGGTAAC	
Oligo B	GCGGATAACAATTTCACACAGGAAACAG	
PCR conditions: http://wmd3.weigelworld.org/downloads/Cloning_of_artificial_microRNAs.pdf
**qPCR primers**		
*PHYB1* F	CAATGCTCTAAGAGGCGTGGA	61⋅C/1 min
*PHYB1* R	CTGGAGCAAGCATTAACCACCA	2-step protocol, 40 cycles
*PHYB2* F	GGAAGGGTGGGTAGAAGTCC	61⋅C/1 min
*PHYB2* R	GGGCAAACAATCCTGAACTC	2-step protocol, 40 cycles
*PHYE* F	GGAGACAAGTGAAGCCTGTGAG	60⋅C/1 min
*PHYE* R	TGCCGTCCTCTATACCTCCAA	2-step protocol, 40 cycles
control F (Solyc03g111090)	CTGACTTCTCAGCAGAACTCCAAT	60–62⋅C/1 min
control R (Solyc03g111090)	TTCACCCTTTTCAATGCTCTTCTC	2-step protocol, 40 cycles

### Transgenic plant generation

The *PHYE* amiRNA transgenic lines were generated through Agrobacterium mediated tissue culture transformation by The Ralph M. Parsons Foundation Plant Transformation Facility, University of California, Davis, One Shields Ave, Davis, CA 95616 using a protocol modified from Fillatti et al. (1987) and propagated in the UC Davis College of Biological Sciences Orchard Park Greenhouse Facility.

### Higher order mutant generation

To generate the higher order phytochrome mutants with the *PHYE* amiRNA, a single insertion homozygous *PHYE* amiRNA was crossed with the *phyA/phyB1/phyB2* triple mutant to generate the heterozygous F1. Single insertion was determined through the segregation ratio of the *PHYE* amiRNA transgene in the T2 generation. Fifty seedlings from each of four independent insertion lines were grown, DNA extracted using a CTAB with chloroform protocol, and genotyped by PCR (see Table 1 for genotyping primers and PCR conditions) for presence or absence of the transgene. Lines with a 3:1 transgene present: transgene absent segregation ratio were determined to be single insertion lines. The F1 was allowed to self to generate the F2 populations segregating for *phyA, phyB1, phyB2*, and the *PHYE* amiRNA transgene. Approximately 300 F2 seeds from this population were grown and genotyped by PCR (see Table 1 for genotyping primers and PCR conditions) to identify progeny that represent the different mutant combinations needed to fully characterize the role of *PHYE* in tomato shade avoidance. These genotypes were *phyB1/PHYE* amiRNA and *phyB2/PHYE* amiRNA doubles, the *phyB1/phyB2/PHYE* amiRNA triple, and the *phyA/phyB1/phyB2/PHYE* amiRNA quadruple. Plants containing the *PHYE* amiRNA transgene and homozygous for the phytochrome mutant combinations of choice were transplanted and moved to the greenhouse to bulk seeds for future experiments.

### Growth measurements in high R:FR and low R:FR light conditions

#### Hypocotyl

Seeds were surface sterilized with 50% household bleach and plated on 0.5x MSMO (Sigma-Aldrich) and 0.7% phytagar (Sigma-Aldrich) in Phytatrays (tomato, Sigma-Aldrich) or round petri dishes (Arabidopsis, Fisher). Plated seeds were kept in the dark (tomato) or dark and 4°C (Arabidopsis) for 2 days before they were moved to constant light conditions set to 34 μE red and 7 μE blue light in an LED chamber for 1 day. LED chambers are custom made and are equipped with Quantum Devices Snap-Lite LED modules, part # SL1515-470-670-735, with peak emissions at 470 nm (blue), 670 nm (red), and 735 nm (far-red). After 1 day, seeds were scored for germination to ensure only seeds with synchronized germination were used. After scoring, the Phytatrays/petri dishes of each genotype were split into two different LED chambers. Far-red light was added to each chamber to bring the red: far-red (R:FR) ratio to 1.5 for high R:FR (R:FR 1.5, 34 μE red, 7 μE blue) or 0.5 for low R:FR to induce the shade avoidance response (R:FR 0.5, 34 μE red, 7 μE blue) in a randomized design in LED chambers. At 10 days post plating, seedlings with synchronized germination were collected onto transparencies, scanned, and hypocotyl lengths were measured from the scanned images using ImageJ (Schneider et al., 2012). This experiment was repeated for a total of two replicates, switching the R:FR treatments for each chamber. An average of 20 plants per genotype/treatment/replicate were measured.

#### Internode

Seeds were surfaced sterilized with 50% household bleach and plated on moist paper towels in Phytatrays (Sigma-Aldrich). Plated seeds were kept in the dark for 2 days and then moved into 16 h light:8 h dark under high R:FR conditions [R:FR 1.5, photosynthetically active radiation (PAR) 100 μE] in the growth chamber. One week after plating, uniform seedlings were transplanted to soil (commercial Sunshine Mix No. 1, Sun Gro Horticulture) in four-inch pots in a randomized block design and placed in either high R:FR (R:FR 1.5, PAR 100 μE) or low R:FR (R:FR 0.5, PAR 100 μE) conditions to induce the shade avoidance response in a randomized block design in the growth chamber. In these chambers light was provided by a mixture of far-red fluorescent bulbs with a maximum emission at 750 nm (Interlectric Corporation, Warren, PA) and cool-white fluorescent bulbs (GE, F48T12-CW-1500).

For the *PHYE* amiRNA characterization, 3, 4, and 5 weeks after planting, the epicotyl and first three internodes were measured with digital calipers (Mitutoyo) to capture organ length over time. Six plants per genotype/treatment were measured.

For the higher order mutant analysis, planting was split into three groups staggered by 1 day to allow proper measurements to be obtained. Five weeks after plating the epicotyl and first three internodes were measured with digital calipers to capture organ length. The higher order mutant growth characterization was repeated for a total of two replicates, switching the high R:FR and low R:FR sides of the growth chamber. An average of eight plants per genotype/treatment/replicate was measured.

#### Light measurements

Measurements to determine LED chamber μE red, μE blue, and R:FR ratio and growth chamber PAR and R:FR ratio were taken with a BLACK-Comet CXR-SR-50 (StellarNet Inc.) Light spectra for high R:FR and low R:FR LED and growth chambers are in Supplemental Figure 1.

### Statistical analyses

#### Wild type vs. double mutants (Figure 2)

For each wild-type or mutant strain a *t*-test was performed to determine if the different light conditions caused a significant difference in length. *P*-values were adjusted for multiple comparisons. Scripts for this analysis are available at https://github.com/MaloofLab/Lavelle-phyE-Frontiers-2016.

#### Wild type vs. single mutants (Figure 4)

A linear mixed effects model was fit using the lme4 (Bates et al, 2013, 2015) and lmerTest (Kuznetsova et al., 2014) packages in R (R Core Team, 2014). Genotype, treatment, and measurement day, along with two and three-way interactions were used as fixed effects and flat was used as a random effect. Scripts for this analysis are available at https://github.com/MaloofLab/Lavelle-phyE-Frontiers-2016.

#### Multiple mutant combinations (Figure 6 and Table 2)

To obtain line means and compare the shade avoidance response between Moneymaker and the various mutant combinations a linear mixed effects model was fit using the lme4 (Bates et al, 2013, 2015) and lmerTest (Kuznetsova et al., 2014) packages in R (R Core Team, 2014). Genotype, treatment, and their interaction were used as fixed effects, and stagger date and replicate experiment number were used as random effects. Additional contrasts were tested using the multcomp package (Hothorn et al., 2008). Scripts for this analysis are available at https://github.com/MaloofLab/Lavelle-phyE-Frontiers-2016.

### Gene expression of *PHYB* family genes in the *PHYE* amiRNA background

Plants were grown as outlined for internode growth experiments. Young leaf issue from Moneymaker and the *PHYE* amiRNA line was harvested and flash frozen in liquid nitrogen approximately 3 weeks after planting. Tissue from three plants per genotype was pooled for each of four biological replicates. The Plant RNeasy kit (Qiagen) was used for RNA extraction. qPCR (see Table 1 for qPCR primers and qPCR conditions) was done using a Bio-Rad iCycler to evaluate the affect the *PHYE* amiRNA has on the transcript levels of *PHYB* family members *PHYB1* Solyc01g059870, *PHYB2* Solyc05g053410, and *PHYE* Solyc02g071260.

### Phylogenetic tree

The phylogenetic analysis of the phytochrome family was done using phylogeny.fr “One-Click” without the use of Gblocks (http://www.phylogeny.fr/index.cgi; Dereeper et al., 2008, 2010). Rice (Ouyang et al., 2007), Medicago (Young et al., 2011), and grape (Jaillon et al., 2007) sequences were obtained from Phytozome (phytozome.org; Goodstein et al., 2012), Arabidopsis sequences were obtained from TAIR (arabidopsis.org; Lamesch et al., 2012), and tomato sequences were obtained from Solgenomics (solgemomics.net; Fernandez-Pozo et al., 2015). Locus identifier and annotation for the sequences used in the analysis are in **Table 3**.

## Results and discussion

### The *phyB1/phyB2* double mutant retains a strong shade avoidance response

**Figure 2:**
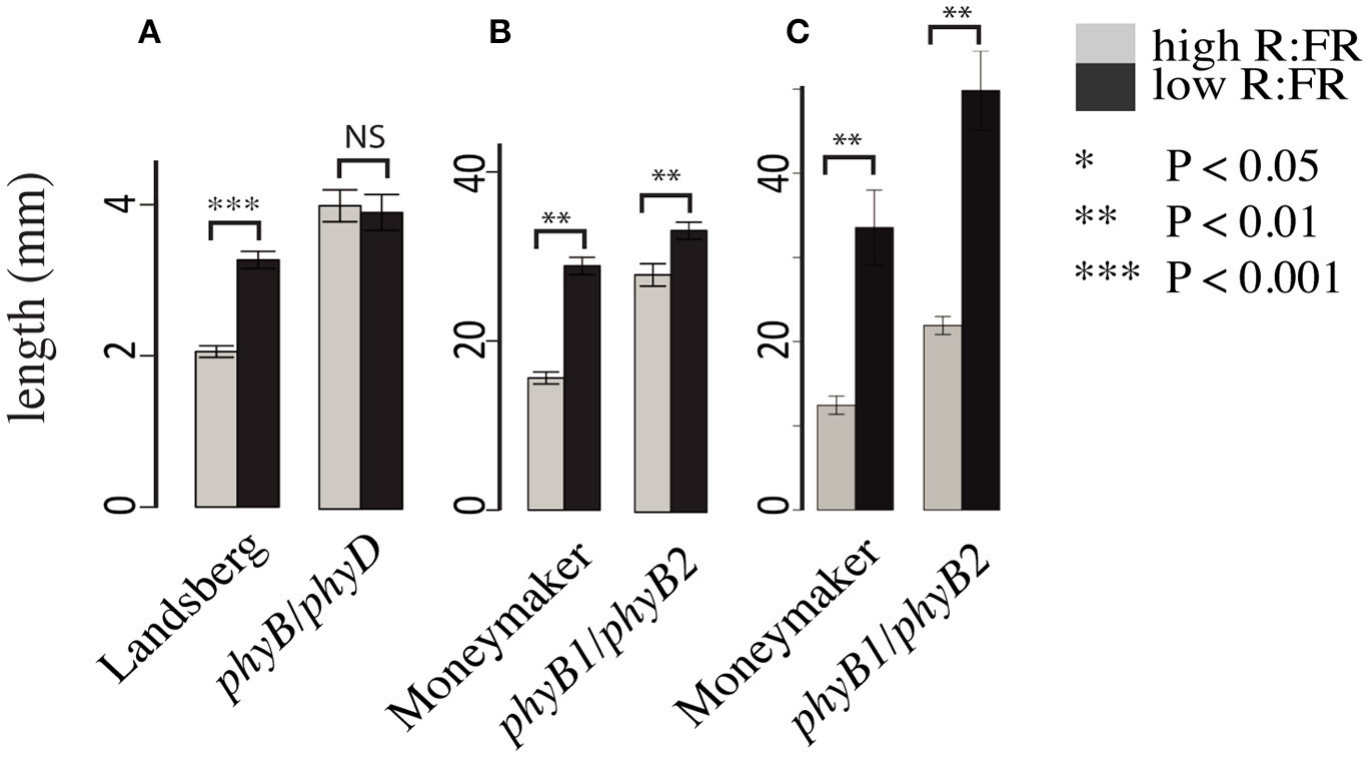
**Growth phenotypes of Arabidopsis and tomato phyB paralog double mutants**. Hypocotyl lengths of **(A)** Arabidopsis *phyB/phyD* double mutants and wild-type Landsberg and **(B)** tomato *phyB1/phyB2* double mutants and wild-type Moneymaker. Measurements were made 10 days post plating on seedlings grown on 0.5x MSMO and 0.7% agar in Phytatrays under constant light in high R:FR and low R:FR conditions. *n* = an average of 20 for each genotype/treatment. Internode length **(C)** of 5 week old *phyB1/phyB2* and Moneymaker plants grown on soil in four-inch pots under long day (16:8) high R:FR and low R:FR conditions in a growth chamber. *n* = 5 plants for each genotype/treatment. The error bars show ±*SE*. ^*^P < 0.05, ^**^P < 0.01, ^***^P < 0.001, NS = not significant by Student's *t*-test.

Consistent with previous work characterizing the phyB family in Arabidopsis (Aukerman et al., 1997; Franklin et al., 2003) and tomato (Weller et al., 2000) we also documented differences between the Arabidopsis and tomato phytochrome families. Most strikingly, the Arabidopsis *phyB/phyD* double mutant does not show a hypocotyl elongation shade avoidance response (Figure 2A) while the analogous tomato *phyB1/phyB2* double mutant does retain a hypocotyl shade avoidance response (Figure 2B) and has internodes that are strongly shade responsive (Figure 2C). Based on the strong shade avoidance response in the tomato *phyB1/phyB2* double mutant we hypothesized that an additional phytochrome, likely the third member of the tomato *PHYB* family, *PHYE*, likely plays a role in the tomato shade avoidance response.

### *PHYE* mRNA is reduced 50% in the *PHYE* amiRNA transgenic line while expression of *PHYB* family members *PHYB1* and *PHYB2* are unaffected

**Figure 3:**
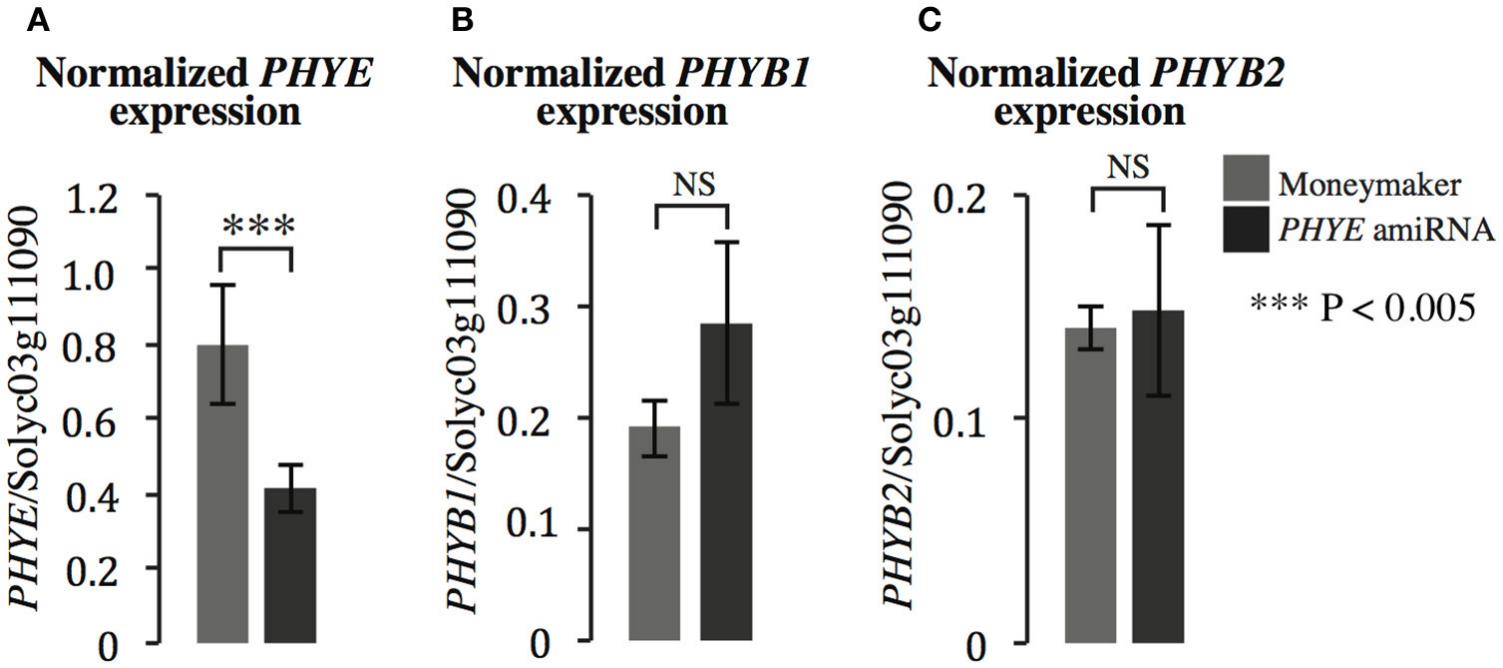
**Gene expression of ***PHYB*** subfamily members (A) ***PHYE***, (B) ***PHYB1***, and (C) ***PHYB2*** in Moneymaker and the ***PHYE*** amiRNA transgenic line**. Leaf tissue was collected from 3-week old transgenic *PHYE* amiRNA and wild type Moneymaker plants grown on soil in four-inch pots under long day (16:8) high R:FR conditions in a growth chamber. *n* = 4 biological replicates, each consisting of three individuals for each genotype. The error bars show ±*SE.* ^***^P < 0.005, NS = not significant by Student's *t*-test.

In order to investigate the role of *PHYE* in internode elongation and shade avoidance, an amiRNA was designed to target *PHYE* using the WMD3-Web MicroRNA Designer (http://wmd3.weigelworld.org/cgi-bin/webapp.cgi; Schwab et al., 2006) and cloned into a binary vector under the control of the constitutive 35S promoter. The construct was transformed into Moneymaker and transformants were screened for single insertion homozygous lines in the T3 generation. In order to demonstrate that the *PHYE* amiRNA is functional and specific to the target of interest, gene expression of the tomato phyB subfamily members (*PHYB1, PHYB2*, and *PHYE*) were assayed by qPCR in the *PHYE* amiRNA line. As expected, the *PHYE* amiRNA does reduce expression of *PHYE* mRNA (Figure 3A) and does not target related family members *PHYB1* (Figure 3B) and *PHYB2* (Figure 3C). The results show that *PHYE* mRNA is reduced about 50% in the transgenic line while expression levels of the other family members are unchanged in the amiRNA line compared to their expression in wild type Moneymaker.

### *PHYE* amiRNA transgenic plants have a wild type phenotype

**Figure 4:**
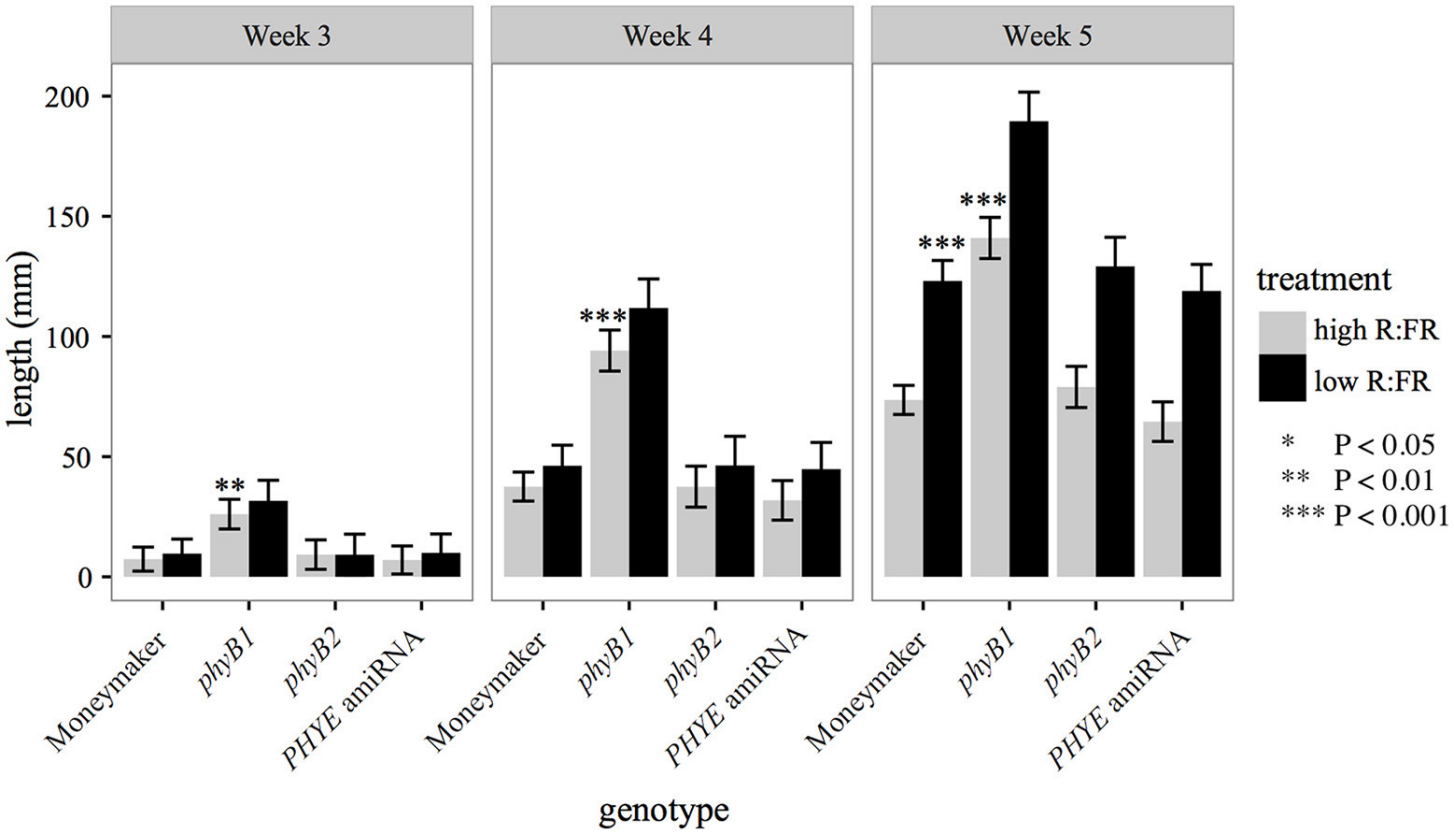
**Growth characteristics of phyB family single mutants and amiRNA in high R:FR and in low R:FR**. Combined epicotyl, internode 1, and internode 2 length of 3, 4, and 5 week-old *phyB1, phyB2, PHYE* amiRNA, and Moneymaker plants grown on soil in four-inch pots under long day (16:8) high R:FR and low R:FR conditions. *n* = 6 for each genotype/treatment. The error bars show ±*SE. phyE* was not significantly different than wild type in high R:FR or low R:FR at any time point (*P* > 0.25). Asterisks for “high R:FR” bars indicate that a mutant was significantly different than Moneymaker in high R:FR at that time. Asterisks for “low R:FR” bars indicated that a mutant had a significantly different *response* to shade as compared to wild type at that time. Asterisks for Moneymaker “low R:FR” are for the high R:FR vs. low R:FR comparison Moneymaker for that time point. ^*^P < 0.05, ^**^P < 0.01, ^***^P < 0.001 by a linear mixed-effects model.

The *PHYE* amiRNA line, previously characterized *phyB1* (Lazarova G. I. et al., 1998a) and *phyB2* (Weller et al., 2000) null mutants, and wild-type Moneymaker were characterized for the internode elongation shade avoidance response over time under long day high R:FR and low R:FR to induce the shade avoidance response in a growth chamber. Like *phyB2* mutants, *PHYE* amiRNA plants have wild-type internode lengths in both high R:FR and low R:FR (Figure 4). In contrast the *phyB1* mutant plants have elongated internodes in high R:FR but a wild-type response to shade (Figure 4). These similarities are especially apparent at 5 weeks of age as the *phyB2, PHYE* amiRNA, and wild-type Moneymaker are all indistinguishable from each other for internode length in high R:FR and response to shade. These results suggest that like phyB2, phyE is not required for wild-type shade avoidance in the presence of other phyB family members; it is possible, however, that since some *PHYE* transcript remains in the *PHYE* amiRNA line, a true *phyE* null monogenic mutant would show an elongated internode length phenotype or reduced shade avoidance response. This finding is consistent with Arabidopsis phytochrome family, as the monogenic Arabidopsis *phyB* mutant is the only phyB subfamily member to display a defective elongation phenotype. The Arabidopsis *phyD* (Devlin et al., 1999) and *phyE* (Devlin et al., 1998) monogenic mutants both have a wild-type growth phenotype.

### *PHYE* is redundant for epicotyl and internode elongation and shade avoidance

**Figure 5:**
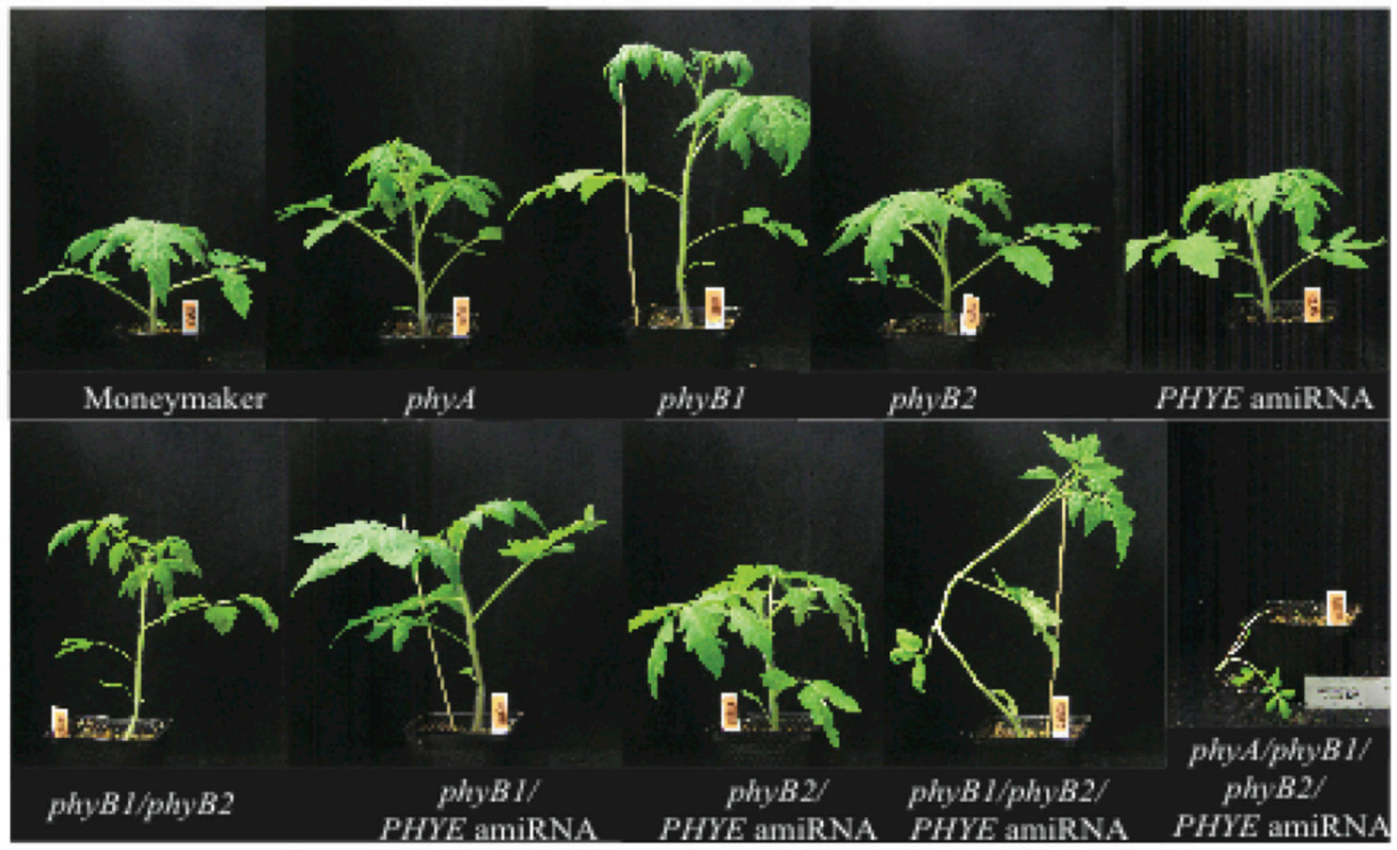
**Growth phenotypes of 5-week old high R:FR grown Moneymaker, single mutants ***phyA***, ***phyB1***, ***phyB2***, ***PHYE*** amiRNA, double mutants ***phyB1/phyB2***, ***phyB1/PHYE*** amiRNA, ***phyB2/PHYE*** amiRNA, triple mutant ***phyB1/phyB2/PHYE*** amiRNA, and quadruple mutant ***phyA/phyB1/phyB2/PHYE*** amiRNA**.

To fully investigate the role of *PHYE* in the control of epicotyl and internode length and internode elongation response to shade, higher order mutants were generated by crossing the *PHYE* amiRNA transgenic line with the *phyA/phyB1/phyB2* triple mutant. The heterozygous F1 progeny of this cross was then grown and allowed to self-pollinate to generate an F2 population segregating for *phyA, phyB1, phyB2*, and the *PHYE* amiRNA transgene. Individuals from this population were genotyped to identify the lines that represent the different double, triple, and quadruple mutant combinations needed to fully characterize the role of *PHYE* in elongation and shade avoidance. Once the homozygous higher order mutant lines were obtained, epicotyl length, internode length, and increased elongation in response to shade were characterized in phyB subfamily double mutants *phyB1/phyB2, phyB1/PHYE* amiRNA, and *phyB2/PHYE* amiRNA, the phyB subfamily triple mutant *phyB1/phyB2/PHYE* amiRNA, and quadruple mutant *phyA/phyB1/phyB2/PHYE* amiRNA under long day high R:FR and shade conditions in a growth chamber (Figure 5).

**Figure 6:**
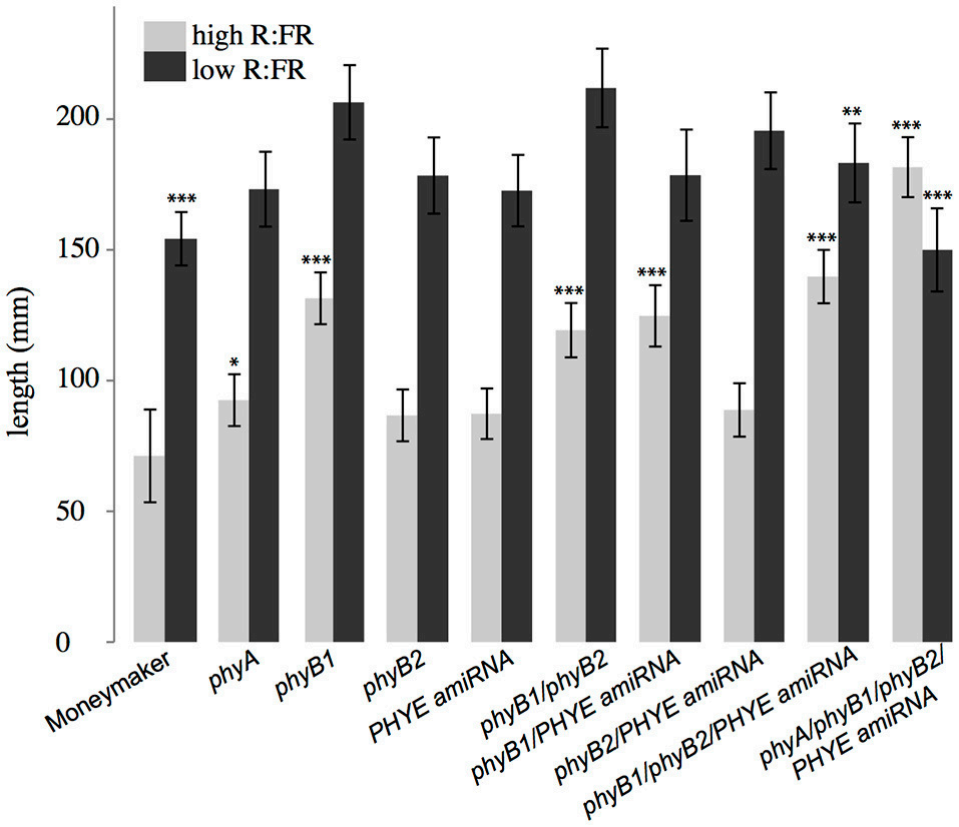
**Growth phenotypes of higher order phytochrome mutants**. Total length of epicotyl, internode 1, and internode 2 of 5-week old Moneymaker and phytochrome single, double triple, and quadruple mutant plants grown on soil in four-inch pots under long day (16:8) high R:FR and low R:FR conditions in a growth chamber. *n* = an average of eight plants for each genotype/treatment/replicate. The error bars show ±*SE.* Asterisks for mutant “high R:FR” bars indicate that a mutant was significantly different than Moneymaker in high R:FR. Asterisks for mutant “low R:FR” bars indicated that a mutant had a significantly different *response* to shade as compared to wild type at that time. Asterisks for Moneymaker “low R:FR” are for the high R:FR vs. low R:FR comparison in Moneymaker. *P*-values for additional contrasts are provided in Table 3. ^*^P < 0.05, ^**^P < 0.01, ^***^P < 0.001 by a linear mixed-effects model.

Double mutant analysis of the *phyB1/phyB2, phyB1/PHYE* amiRNA, and *phyB2/PHYE* amiRNA lines revealed overlapping roles in the tomato *PHYB* subfamily. Interestingly, like their respective individual single mutants, both internode length and shade avoidance in the *phyB2/PHYE* amiRNA double mutant are not significantly different from wild type (Figure 6), further suggesting that phyB1 is sufficient for wild-type internode growth and shade avoidance under these conditions. The *phyB1* single mutant and *phyB1/PHYE* amiRNA double mutant are not significantly different from each other with respect to internode length in high R:FR but *phyB1/PHYE* amiRNA is less responsive than the *phyB1* single for internode elongation in response to shade (Figure 6 and Table 2) indicating that phyB2 and/or residual phyE provide some shade responsiveness in these lines. Finally, the *phyB1/phyB2* double mutant was significantly taller than wild type in high R:FR but did not show a significantly different response to shade than wild type (Figure 6). Although the double mutant analysis does not provide direct information about the role of *PHYE* in internode elongation or shade avoidance, the shade responsiveness of the *phyB1/phyB2* double mutant shows that an additional gene or genes are involved in the control of shade avoidance. *PHYE* is the likely candidate as the third member of the *PHYB* subfamily that has been shown to play a central role in shade avoidance.

**Table 2:** **Additional statistical comparisons between mutant combinations**.

**Contrast**	***p*-value**
**GENOTYPE EFFECTS**	
*phyB1 PHYE* amiRNA vs. *phyB1*	0.236
*phyB2 PHYE* amiRNA vs. *phyB2*	0.100
*phyB1/phyB2/PHYE* amiRNA vs. *phyB1/phyB2*	0.691
*phyA/phyB1/phyB2/PHYE* amiRNA vs. *phyB1/phyB2/PHYE* amiRNA	1.78e-15
**GENOTYPE BY TREATMENT INTERACTION EFFECTS**	
*phyB1 PHYE* amiRNA vs. *phyB1*	0.883
*phyB2 PHYE* amiRNA vs. *phyB2*	0.924
*phyB1/phyB2/PHYE* amiRNA vs. *phyB1/phyB2*	0.010
*phyA/ phyB1/phyB2/PHYE* amiRNA vs. *phyB1/phyB2/PHYE* amiRNA	4.32e-05

*Several additional specific contrasts were made between different genotypes. Comparisons were made using the multcomp package (Hothorn et al., 2008) in R (R Core Team, 2014), evaluating a linear mixed effects model fit by lme4 (Bates et al, 2013, 2015). The “genotype effects” section reports the p-values for whether or not the internode lengths were different in high R:FR for the specified genotypes. The “Genotype by treatment interaction effects” section reports the p-values for whether the response to shade was different between the specified genotypes*.

**Table 3:** **Sequences for phylogenetic analysis**.

**Species**	**Locus identifier**	**Annotation**
Arabidopsis	AT1G09570	phytochrome A
	AT2G18790	phytochrome B
	AT5G35840	phytochrome C
	AT4G16250	phytochrome D
	AT4G18130	phytochrome E
Tomato	Solyc10g044670	phytochrome A
	Solyc01g059870	phytochrome B1
	Solyc05g053410	phytochrome B2
	Solyc02g071260	phytochrome E
	Solyc07g045480	phytochrome F (phytochrome C)
Grape	GSVIVG01031354001	phytochrome A
	GSVIVG01034989001	phytochrome B
	GSVIVG01030081001	Two-component sensor histidine kinase
	GSVIVG01021381001	phytochrome E
Medicago	Medtr1g085160	phytochrome A
	Medtr2g034040	phytochrome B
	Medtr2g049520	phytochrome E
Rice	Os03g51030	phytochrome A
	Os03g19590	phytochrome B
	Os03g54084	phytochrome C

### Triple and quadruple mutants reveal a role for phyE in internode elongation and shade avoidance

Although neither single nor double mutant analysis uncovered a specific role for *PHYE* in either internode elongation or shade avoidance, characterization of the *phyB1/phyB2//PHYE* amiRNA triple mutant revealed a role for *PHYE* in shade avoidance. While the *phyB1/phyB2/PHYE* amiRNA triple mutant had a similar internode length to the *phyB1* single and *phyB1* double mutants, shade avoidance is significantly reduced in the *phyB1/phyB2/PHYE* amiRNA triple mutant as compared to wild type or the *phyB1/phyB2* double mutant (*p* = 0.009, 0.01, respectively; Figure 6 and Table 2). These results show that *PHYE* is required for shade avoidance in the absence of *PHYB1* and *PHYB2*, indicating a distinct role for *PHYE* in the internode elongation response to shade. Beyond the tomato *PHYB* subfamily, the *phyA/phyB1/phyB2/PHYE* amiRNA quadruple mutant shows both significant internode elongation and loss of a shade avoidance response.

## Conclusions

The internode length and internode shade avoidance response of higher order tomato *PHYB* subfamily mutants highlight both similarities and differences between the tomato and Arabidopsis phytochrome families. Tomato *PHYB1*, like Arabidopsis *PHYB*, is the dominant phytochrome in both internode elongation and shade avoidance while tomato *PHYB2* and *PHYE* and Arabidopsis *PHYD* and *PHYE* play largely redundant roles as monogenic mutants in any of the genes display a wild-type phenotype. Although it is possible that *PHYE* plays a larger role in both internode elongation and shade avoidance that cannot be seen due to the incomplete knockdown of *PHYE* mRNA in the *PHYE* amiRNA line, like Arabidopsis *PHYD* and *PHYE*, the role of tomato *PHYB2* and *PHYE* are more readily seen in the tomato *phyB1* background. There is a clear role for *PHYE* in tomato shade avoidance. The most interesting difference between the tomato and Arabidopsis phytochrome families comes with the comparison between the shade responsive tomato *phyB1/phyB2* double mutant and non-shade responsive *phyB1/phyB2/ PHYE* amiRNA triple mutant. This result clearly outlines a role for *PHYE* in the internode elongation response for shade.

## Author contributions

AS and JM conceived and designed the experiments; AS and JM wrote the article; AS, LH, and JM conducted the experiments, analyzed data, and contributed to the study design.

## Funding

This work was supported in part by the Elsie Taylor Stocking Memorial Fellowship awarded to AS in 2013, by NSF grant IOS-0820854, by United States Department of Agriculture NIFA project (http://www.csrees.usda.gov/; CA-D-PLB-7226-H) to JM, and by internal UC Davis funds.

### Conflict of interest statement

The authors declare that the research was conducted in the absence of any commercial or financial relationships that could be construed as a potential conflict of interest.
