# Supplementary figures and images for: Tomato phyE Is Required for Shade Avoidance in the Absence of phyB1 and phyB2
Source: Front Plant Sci. 2016 Sep 16;7:1275. doi: 10.3389/fpls.2016.01275 (PMC5025638; doi:10.3389/fpls.2016.01275)

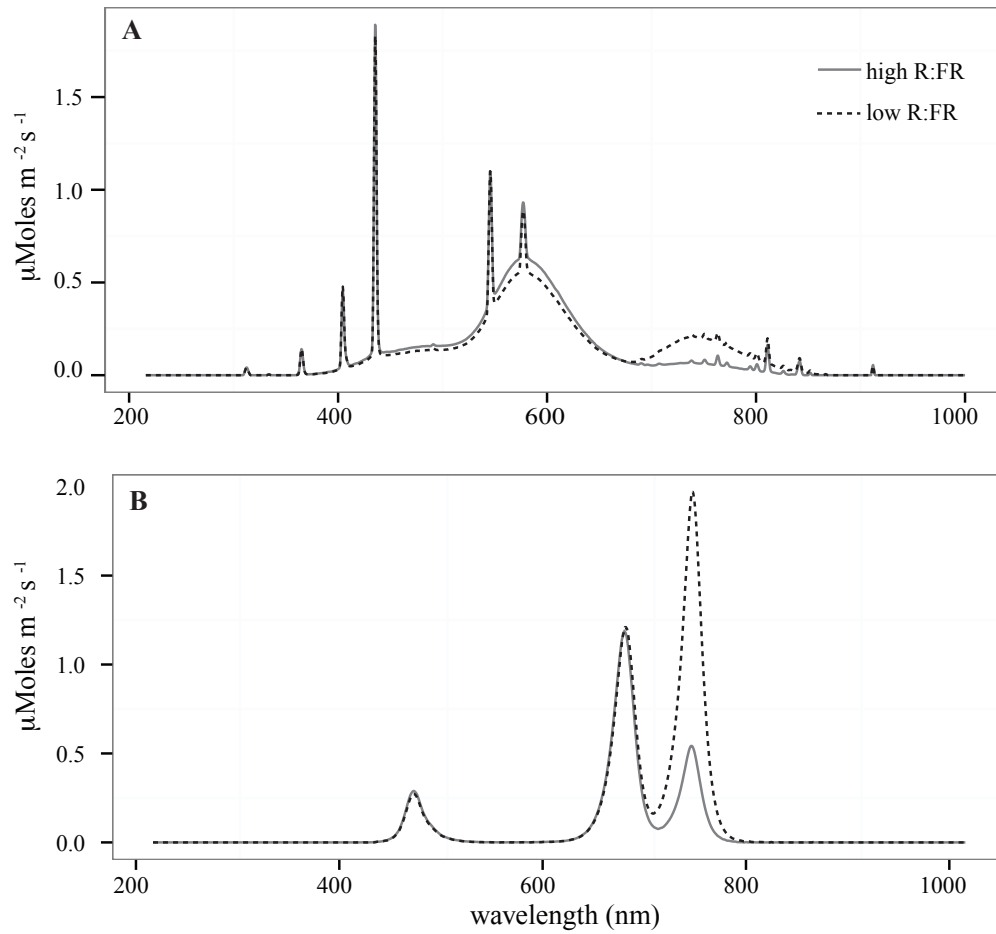

Supplemental figure 1. Light spectrum of growth chamber (A) and LED chamber (B) high R:FR and low R:FR.

Supplement: Supplementary file 1 [file Image1.PDF]
